# Complete genome sequences of two actinomycetes containing abyssomicin-like gene clusters: *Kutzneria buriramensis* DSM 45791 and *Streptomyces* sp. NL15-2K

**DOI:** 10.1128/mra.01158-24

**Published:** 2025-01-15

**Authors:** Sam E. Williams, David Faurdal, Tue S. Jørgensen, Catherine R. Back, Lynden D. Rooms, Martin A. Hayes, Christine L. Willis, James E. M. Stach, Paul Curnow, Paul R. Race

**Affiliations:** 1 Novo Nordisk Foundation Center for Biosustainability, Technical University of Denmark, Lyngby, Denmark; 2 School of Biochemistry, University of Bristol, Bristol, United Kingdom; 3 Discovery Sciences, Biopharmaceuticals R&D, AstraZeneca, Sweden, Mölndal; 4 School of Chemistry, University of Bristol, Bristol, United Kingdom; 5 School of Natural and Environmental Sciences, Newcastle University, Newcastle upon Tyne, United Kingdom; Portland State University, Portland, Oregon, USA

**Keywords:** actinomycetes, genomes, abyssomicin, *Streptomyces *NL15-2K, *Kutzneria buriramensis*, genome mining for antibiotics, actinomycetes antibiotic discovery, biosynthetic gene clusters, Oxford Nanopore Sequencing, secondary metabolism

## Abstract

Here, we report the resequencing, assembly, and annotation of two actinomycete genomes containing abyssomicin gene clusters. *Kutzneria buriramensis* DSM 45791 with a circular chromosome of 11,681,598 bp and 4 circular plasmids (14,175–207,548 bp) and *Streptomyces* sp. NL15-2K with a 12,368,159 bp linear genome and circular plasmid (11,584 bp).

## ANNOUNCEMENT

The abyssomicin family of spirotetronate natural products includes potent antibacterial and antitumor agents, whose biosynthesis incorporates uncommon biosynthetic transformations, including Diels-Alderase mediated cycloadditions (1). In search of abyssomicins, we identified two promising but fragmented biosynthetic gene clusters (BGCs) with incomplete assembly: *Streptomyces* NL15-2K, comprising 346 contigs (GCF_003851625.1) and *Kutzneria buriramensis* DSM 45791, comprising 65 contigs (GCF_003387475.1). *Streptomyces* NL15-2K was isolated from forest soil in Vancouver, Canada (2) and studied for lignin degradation (3). *K. buriramensis* DSM 45791 was isolated from forest soil in Buriram, Thailand (4). We resequenced both strains, obtaining *Streptomyces* NL15-2K gDNA (2) from Prof. Motohiro Nishimura (Yasuda Women’s University) and *K. buriramensis* from The Leibniz Institute DSMZ. DNA was extracted using Genomic Tip 20/G (QIAGEN) adapted for actinobacteria (5).

Rapid-barcoding libraries (SQK-RBK114-24; Oxford Nanopore Technologies) were sequenced on a MinION with an R10.4 flow cell. Raw sequencing data were demultiplexed and basecalled using Guppy (v6.4.8) high-accuracy model. Illumina, 2 × 250 bp paired-end sequencing was performed on a NovaSeq 6000 (Illumina) using the Nextera XT Library Prep Kit as a service by MicrobesNG (https://microbesng.com/).

All tools were run with default parameters unless otherwise specified. Nanopore reads were trimmed using Filtlong (0.2.1) with min_length 1000 and keep_percent 95 (*K. buriramensis*) and 67.5 (*Streptomyces* NL15-2K). Resulting N50 values were 12,372 bp for *K. buriramensis* and 10,039 bp for *Streptomyces* NL15-2K. Illumina reads were trimmed with Trimmomatic (v0.30) with a Q15 cut-off (6).

Genome assembly for *K. buriramensis* used Trycycler (v0.5.4) (7) with 12 read subsets (read depth 52.4×). Four subsets were assembled by Raven (v1.8.0) (8), Flye (nano-hq) (v2.9.1) (9), or Canu (GenomeSize = 12 m) (v2.1.1) (10). Assemblies were reconciled into five contigs with Trycycler. Streptomyces NL15-2K was assembled using Flye (nano-hq) (v2.9.3), followed by the NPGM-contigger to resolve the terminal inverted repeats (TIR) at the chromosomal ends (DOI 10.11583/DTU.25133828). Illumina reads were aligned with BWA-MEM2 (v2.2.1) (11) and polished with polypolish (v0.6.0) (12) (Table 1).

**TABLE 1:** Assembly statistics for sequenced genomes and reads after quality filtering steps^*d*^

Strain	Assembly method	ONT read count (base pairs)	ONT read depth	Contigs per assembly	Final contigs	Illumina paired read count	Illumina read depth^*b*^	Prepolishing accuracy^*c*^	Plasmids
*Streptomyces* NL15-2K	Flye + NPGM-Contigger	64,840 (606,393,990 bp)	48.9×	3^*a*^	2	2,188,558	75.7×	99.9934%	1
*Kutzneria buriramensis* DSM 45791	Trycycler	178,781 (1,370,129,713 bp)	113.1×	5–13	5	2,642,996	84.4×	99.9981%	4

^*a*^ Number of contigs before applying the contigger.

^*b*^ Illumina read depth is mean depth across the chromosome contig.

^*c*^ Prepolishing accuracy estimated by polypolish.

^*d*^ ONT, Oxford Nanopore Technologies.

The genome of *Streptomyces* NL15-2K has a G + C content of 70.27%, consisting of a 12,368,159 bp linear chromosome with 38 kb TIR and a 11,584 bp circular plasmid. The linear replicon was determined to be complete by mapping all reads against the chromosome and inspecting terminally mapped reads. Final genome coverage was 48.9× ONT and 75.7× Illumina. BUSCO (v5.4.6) (13) analysis (lineage: actinobacteria_class_odb10) showed 100% complete core genes. Phylogenomic analysis (https://tygs.dsmz.de) (14) suggested NL15-2K represents a distinct species with 33.4% DNA-DNA hybridization to its closest relative. NCBI PGAP v.6.7 (15) identified 10,982 genes, 6 complete rRNAs, and 74 tRNAs.

*K. buriramensis* DSM 45791 has a G + C content of 70.34% across a 11,681,598 bp circular chromosome and 4 plasmids (14,175 bp, 51,178 bp, 56,886 bp, 207,548 bp). Circular chromosome overlaps were resolved with Trycycler, with *dnaA* at the sequence start. Final genome coverage was 113.1× ONT and 84.4× Illumina. BUSCO analysis showed 99.7% complete core genes. NCBI PGAP predicted 11,057 genes, 3 complete rRNAs, 55 tRNAs, and 3 CRISPR arrays.

AntiSMASH 7.1.0 (16) identified 41 BGCs in *Streptomyces* NL15-2K and 51 BGCs in *K. buriramensis*, indicating significant biosynthetic potential.

## Data Availability

All data are available under BioProject PRJNA995553. Raw reads are deposited at SRA with accession SRX21046384–SRX21046385 (Illumina) and SRX21046382–SRX21046383 (ONT). The assembly accession numbers for *K. buriramensis* DSM 45791 are CP144375.1–CP144379.1 and for Streptomyces NL15-2K are CP130630.2 and CP130632.2.
